# Revealing Ultrafast Charge-Carrier Thermalization in Tin-Iodide Perovskites through Novel Pump–Push–Probe Terahertz Spectroscopy

**DOI:** 10.1021/acsphotonics.1c00763

**Published:** 2021-08-09

**Authors:** Aleksander M. Ulatowski, Michael D. Farrar, Henry J. Snaith, Michael B. Johnston, Laura M. Herz

**Affiliations:** Department of Physics, University of Oxford, Clarendon Laboratory, Parks Road, Oxford OX1 3PU, U.K.

**Keywords:** ultrafast spectroscopy, visible-pump—IR-push—THz-probe, tin-triiodide perovskites, hot-carrier cooling

## Abstract

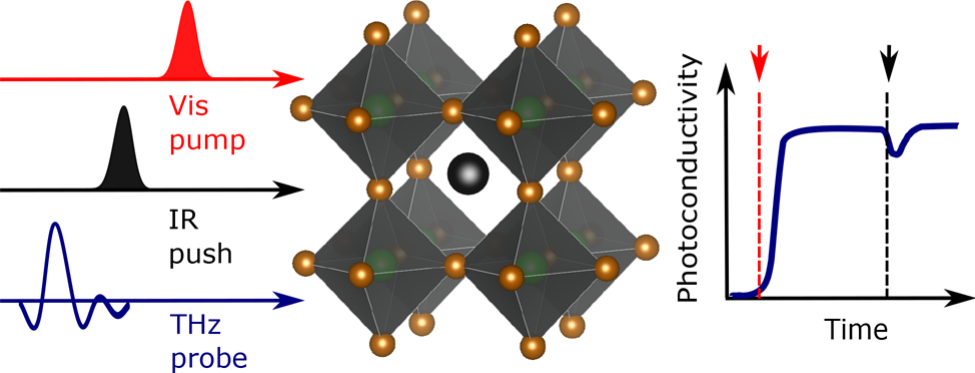

Tin-iodide perovskites are an important group of semiconductors for photovoltaic applications, promising higher intrinsic charge-carrier mobilities and lower toxicity than their lead-based counterparts. Controllable tin vacancy formation and the ensuing hole doping provide interesting opportunities to investigate dynamic intraband transitions of charge carriers in these materials. Here, we present for the first time an experimental implementation of a novel Optical-Pump–IR-Push–THz-Probe spectroscopic technique and demonstrate its suitability to investigate the intraband relaxation dynamics of charge carriers brought into nonequilibrium by an infrared “push” pulse. We observe a push-induced decrease of terahertz conductivity for both chemically- and photodoped FA_0.83_Cs_0.17_SnI_3_ thin films and show that these effects derive from stimulated THz emission. We use this technique to reveal that newly photogenerated charge carriers relax within the bands of FA_0.83_Cs_0.17_SnI_3_ on a subpicosecond time scale when a large, already fully thermalized (cold) population of charge-carriers is present. Such rapid dissipation of the initial charge-carrier energy suggests that the propensity of tin halide perovskites toward unintentional self-doping resulting from tin vacancy formation makes these materials less suited to implementation in hot-carrier solar cells than their lead-based counterparts.

Metal-halide perovskites have received significant attention from the scientific community over the past decade because of their excellent optoelectronic properties, including high absorption coefficients,^1^ long charge-carrier diffusion lengths,^2−4^ and low exciton binding energies,^5−7^ which make them well suited to photovoltaic (PV) applications. Since the first implementation of lead-free perovskites in PV,^8,9^ intense research has investigated the properties of tin-iodide perovskites, which promise higher fundamental charge-carrier mobilities than archetypal MAPbI_3_^10,11^ as well as an absence of toxic lead. However, the most significant challenge for tin-containing perovskites is the formation of tin vacancies and iodide interstitials,^12−15^ which induce hole self-doping of the material and promote oxidation of Sn^2+^ to Sn^4+^, commonly reported in the literature.^16,17^ This self-doping effect increases the recombination rate of photoexcited charge carriers and reduces the charge-carrier mobility of the perovskite through the introduction of additional carrier-scattering processes.^18,19^ Several additives have been shown to reduce tin vacancy formation and tin oxidation,^15,20−25^ which has led to efficient tin-iodide perovskite PV devices. Interestingly, these investigations have also yielded perovskite semiconductors with controllable hole doping levels,^18,19,25^ which is still an elusive goal in most lead halide perovskites because of their low barriers to ionic migration and the associated phase segregation.^26,27^

In addition to their advantageous properties of nontoxicity and high charge-carrier mobility, tin-halide perovskites have also been postulated to exhibit slow cooling of hot charge carriers following above-gap excitation, lasting tens of picoseconds^28^ to nanoseconds.^29,30^ Slow carrier cooling times may potentially allow for more efficient harvesting of the energy of hot carriers^31^ in suitably designed hot-carrier solar cell architectures, with efficiencies that could exceed the standard efficiency limit for a device operating in thermal equilibrium.^32^ However, to assess the potential of this technology, the full dynamic energy exchange and loss processes of hot carriers needs to be understood as they ultimately define the intrinsic voltage losses that could be averted in a nonstandard device geometry. Following above-bandgap photoexcitation, charge carriers quickly exchange their energy through carrier–carrier scattering, leading to a broad Maxwell–Boltzmann distribution of charge carriers in their respective bands that is characterized by a temperature *T*_c_, higher than that of the lattice.^33^ This so-called thermalization process has been investigated in lead-halide perovskites through two-dimensional electronic spectroscopy and was shown to last tens of femtoseconds. An increased charge-carrier concentration and excess energy of the excitation was shown to accelerate the dynamics because of the concomitant increase in carrier–carrier scattering rates.^34^ Following this thermalization process, charge carriers cool to the lattice temperature in a slower process dominated by interactions with optical and acoustic phonons. During this process, the excess energy of carriers in the high-energy tail of the Maxwell–Boltzmann distribution can be extracted in a suitably designed hot-carrier device,^31^ indicating that materials with slow carrier cooling would allow for higher efficiency of this energy harvesting process. Such charge-carrier cooling times have been investigated for metal-halide perovskites with ultrafast photoluminescence and transient absorption spectroscopy, with reported time scales ranging from hundreds of femtoseconds^35^ to tens of picoseconds,^28^ with some reports of nanoseconds lifetimes.^29,30,36^ Importantly, cooling time scales were prolonged by higher charge-carrier densities, and by higher excess excitation energies because of the phonon bottleneck effect, for which a buildup of hot phonons prevents effective transfer of thermal energy from charge carriers to the surrounding lattice.

While the above-mentioned investigations of charge-carrier thermalization and subsequent cooling dynamics have provided interesting fundamental insights, they unfortunately do not fully reflect the intraband relaxation processes encountered in a photovoltaic cell operating under solar illumination conditions. The majority of studies investigating hot-carrier relaxation dynamics have utilized short excitation pulses with a relatively narrow spectrum (compared with sun light) to create a substantially larger population of nonequilibrium charge carriers than present in a photovoltaic device during operation, with a sharp energy distribution above the bandgap.^28−30,34,36^ This scenario is fundamentally different to hot-carrier relaxation processes in photovoltaic devices, in which any charge carrier newly created with excess energy above the band gap encounters an already present thermalized population of cooled carriers that had been created by the continuous, broad-band illumination of sunlight some time ago. As pointed out by Richter et al.,^34^ newly created nonequilibrium charge-carrier pairs may therefore scatter effectively with an already present ocean of cold electrons and holes, meaning that their excess energy will be transferred to a larger population of carriers through a fast thermalization processes, reducing the overall temperature of the carrier gas much quicker than any study using a narrowband resonant excitation would imply. Such interactions of hot carriers with cold carriers have been studied in perovskites only at very high nonequilibrium photoexcitation densities,^37−40^ for which relaxation rates may still be dominated by carrier-phonon interactions, slowed down by the hot phonon bottleneck effect. Moreover, no such studies have been performed on tin-iodide perovskites, in which the inevitable self-doping and the ensuing thermalized hole population in the valence band can exacerbate scattering processes between hot and cold carriers. Such materials with a high propensity toward self-doping therefore require a particularly careful assessment of their suitability for hot-carrier extraction devices, as the very large population of cold holes at the top of the valence band can effectively absorb the energy of a relatively small population of hot carriers photoexcited by solar illumination. Because of the large imbalance between the charge-carrier density injected under solar illumination conditions (∼10^16^ cm^–3^)^4^ and the background doping density typically present in tin iodide perovskites (∼10^17^–10^20^ cm^–3^),^41^ rapid thermalization of photoinjected charge carriers would be expected to only marginally increase the temperature of the carrier gas available for above-bandgap extraction. Determining the rate at which such interchange occurs is therefore important for the assessment of tin iodide perovskites for hot-carrier photovoltaic applications.

In this study, we investigate the intraband relaxation processes in FA_0.83_Cs_0.17_SnI_3_ thin films, for the first time using a novel ultrafast pump-push-probe (PPP) technique. We exploit the controllable level of doping in tin-iodide perovskites through tin-fluoride addition^18,19^ to examine the impact of a cold charge-carrier gas on the relaxation dynamics of hot carriers. We show that our newly developed Optical-Pump–IR-Push–THz-Probe spectroscopy is a powerful tool that can be used to reveal the effects of intrinsic chemical doping and transient photodoping on the terahertz (THz) conductivity following the illumination of the material with an ultrafast sub-bandgap, infrared (IR) excitation pulse. A transient decrease of conductivity upon this IR excitation in both photo- and chemically doped materials is reported and associated with intraband photoexcitation of free charge carriers and their subsequent interaction with the THz probe. Such nonequilibrium charge carriers subsequently relax toward the edges of their respective bands, with time scales of hundreds of femtoseconds depending strongly on the excess energy provided by the IR “push” pulse. For the relatively low densities of generated nonequilibrium charge carriers, we find hot-phonon bottleneck effects to be absent, with intraband energy loss deriving mostly from scattering with already present cold electrons and holes, which competes with carrier–phonon scattering. Finally, we investigate the origin of negative THz photoconductivity in a literature context,^42−47^ finding that stimulated THz emission from pushed charge carriers is most likely to be responsible for the observed negative photoconductivity response to intraband excitation.

## Results and Discussion

We start our investigation with a determination of the doping level present in thin films of tin-iodide perovskite using terahertz time-domain spectroscopy (THz-TDS), for which single-cycle THz radiation acts as a noncontact probe of sample conductivity.^48^ The transmission of this radiation through a thin film sample is measured using an optical gating method and, by comparison with the transmission through a bare substrate, the frequency-dependent conductivity of the thin film is calculated,^49,50^ as explained in detail in Section IV A of the Supporting Information.

**Figure 1:**
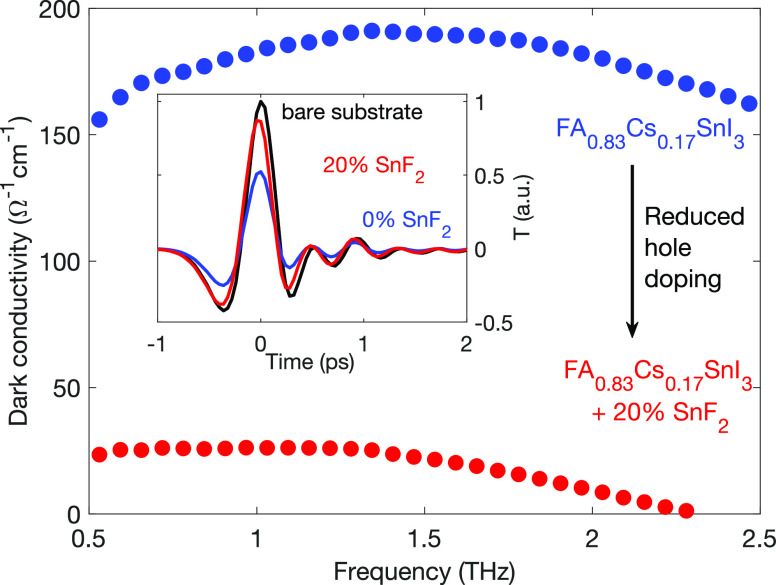
The effect of tin-fluoride addition to FA_0.83_Cs_0.17_SnI_3_ perovskite on the THz dark conductivity spectrum of thin films. Filled blue and red circles show the real part of the THz conductivity in the absence of photoexcitation, for FA_0.83_Cs_0.17_SnI_3_ films fabricated when either no or 20% SnF_2_ had been added to the precursor solution, respectively. The inset displays the corresponding time-domain electric field strength *T* of the transmitted THz probe through the thin films (blue and red lines) and through a bare z-cut quartz substrate (black line).

Figure 1 shows the calculated conductivity spectra in the dark (no photoexcitation) of FA_0.83_Cs_0.17_SnI_3_ thin films for the two cases of either no (blue filled circles) or 20% (red filled circles) of tin fluoride having been added to the precursor solution during deposition (see Section II in the Supporting Information for film processing details). As seen in the figure, the addition of the SnF_2_ significantly reduces the dark conductivity of tin iodide perovskite films by suppressing the presence of tin vacancies and the concomitant hole self-doping of the material.^15,19,20^ We note that when 20% SnF_2_ has been added, the remnant conductivity spectrum mostly reflects contributions from broad optical tin-iodide phonon modes, as discussed previously.^19^ The inset of this figure shows that the high conductivity of the FA_0.83_Cs_0.17_SnI_3_ thin film with no tin-fluoride added leads to a reduction of the electric field strength of the transmitted THz probe, here shown in the time-domain. Upon addition of tin fluoride to the perovskite during deposition, a similar transmission to that of the bare quartz substrate is recovered, indicating negligible conductivity of the thin film. Using an analysis of THz transmission through these thin films,^19,51^ we determine a background doping density of 5 × 10^19^cm^–3^ for the FA_0.83_Cs_0.17_SnI_3_ film without tin-fluoride additive. Existing studies evaluating tin vacancy suppression methods in tin-halide perovskites have found that the minimal doping levels achievable through tin-fluoride addition in these materials are >10^17^cm^–3^.^8,18,20,52^ In our case, in the FA_0.83_Cs_0.17_SnI_3_ thin film fabricated with 20% SnF_2_ addition, the doping concentration falls below the level detectable within our measurement accuracy (∼2 × 10^18^cm^–3^);^19^ therefore, the two samples (with 0% SnF_2_ and 20% SnF_2_ added) represent two convenient scenarios of the highest and lowest hole doping densities attainable in tin-halide perovskites. We note, however, that even in the perovskite film to which 20% SnF_2_ had been added during fabrication, the doping level is high when compared with the density of photoexcited charge carriers typically present under solar illumination (∼10^16^ cm^–3^).^4^

**Figure 2:**
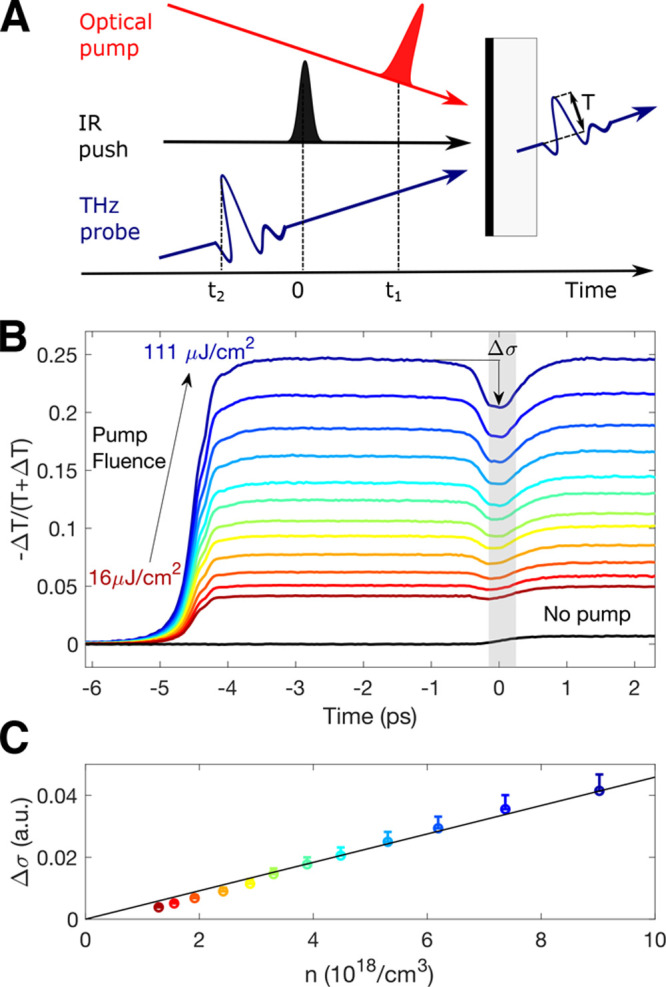
Optical-Pump–IR-Push–THz-Probe (PPP) experiments on a FA_0.83_Cs_0.17_SnI_3_ thin film with low hole doping (20% SnF_2_). (A) schematic diagram of the PPP experiment featuring a thin-film sample deposited on a transparent substrate, photoexcited with an ultrafast laser pulse of above-bandgap photon energy (the “pump”) and subsequently illuminated by an infrared pulse (the “push”) and THz radiation (the “probe”) in order to determine its time-dependent conductivity. (B) Time-dependent photoconductivity transients measured in such an experiment for a FA_0.83_Cs_0.17_SnI_3_ thin film, fabricated with an addition of 20% SnF_2_ to the precursor solution. The negative value of the relative change in THz field amplitude transmission (−Δ*T*/(*T* + Δ*T*)) is proportional to the change in conductivity. The colors of the transients correspond to different fluences of the initial 800 nm wavelength pump photoexcitation, which arrives at time = −5 ps. The IR push pulse of 1450 nm wavelength arrives at time = 0. (C) Dependence of the push-induced conductivity change on the initially photoexcited charge-carrier density *n*. Here, the push-induced decrease in conductivity Δ*σ* is evaluated as the average photoconductivity change recorded over the gray-shaded region shown in (B) from which the average photoconductivity before the push arrival and the effect of push-pulse excitation only (pump beam blocked - black solid line) had been subtracted. The initially generated charge-carrier density *n* has been estimated from the fluence of the pump and absorption data, assuming 100% photon to charge-carrier pair conversion. The error bars represent measurement uncertainty due to laser power drift over the time of the experiment.

We further proceed by probing the dynamics of the transient photoinduced conductivity for FA_0.83_Cs_0.17_SnI_3_ in the presence of high and low hole doping levels. We note that an extension of the above-mentioned THz TDS technique, Optical-Pump–THz-Probe (OPTP) spectroscopy, has been widely used in recent years to investigate charge-carrier dynamics over picosecond-to-nanosecond time scales in perovskite semiconductors.^53−58^ In this type of experiment, a semiconducting perovskite is photoexcited with an ultrafast (tens of fs) optical laser pulse with above-bandgap photon energy, and its photoinduced conductivity subsequently probed with a THz pulse. By using optical delay stages, this method allows mapping of the photoconductivity transient with subpicosecond time resolution, revealing charge-carrier recombination dynamics and mobility.^4^ Here, we report a novel approach that extends this technique by adding an additional ultrafast infrared pulse, a so-called “push”, which induces low-energy optical transitions in an already photoexcited material.^37,39,40^ A schematic diagram of this experiment is shown in Figure 2A, in which a thin film deposited on a quartz substrate is first photoexcited by an optical pump pulse (red), subsequently illuminated with an IR push pulse (black), and finally probed with THz radiation (blue). The timings between the pump and push pulse arrival (*t*_1_) and push and probe arrival (*t*_2_) are controlled with optical delay stages. Crucially, such Optical-Pump–IR-Push–THz-Probe (PPP) experiments allow us to probe intraband transitions, such as those leading to charge carriers being “pushed” higher within their band, and their subsequent relaxation back toward the band edge.

**Figure 3:**
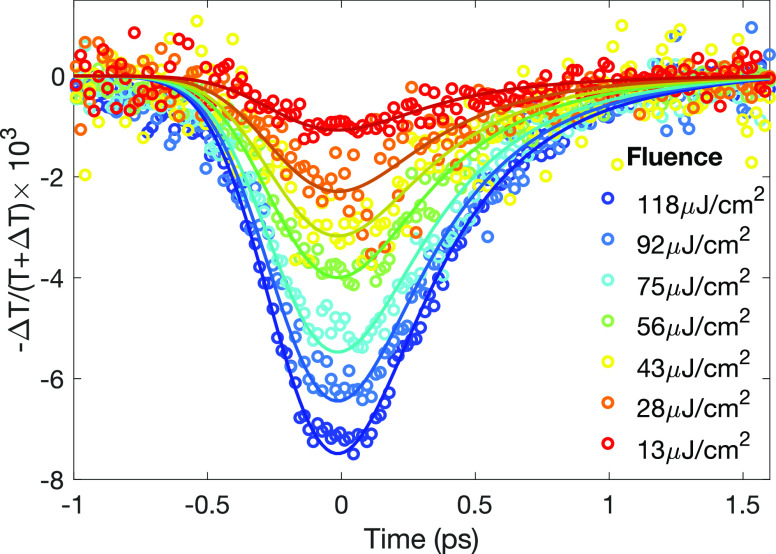
Push-only (pump beam blocked) photoconductivity transients for a FA_0.83_Cs_0.17_SnI_3_ thin film with high hole doping (no tin fluoride additive). Presented transients were induced by sub-bandgap photoexcitation centered at 1390 nm wavelength with excitation fluences indicated in the legend. The relative change in THz field transmission (−Δ*T*/(*T* + Δ*T*)) is proportional to the change in conductivity of the thin film. The empty hoops represent the measured photoconductivity data, and the solid lines show fits of a monoexponential decay convoluted with Gaussian broadening. The standard deviation of the Gaussian (representing system resolution) was determined as *s* = 200 fs, as described in Section VI of the Supporting Information. The fit to the highest fluence data yields a decay time of τ = 357 fs ±10 fs for the relaxation process. This decay lifetime is then fixed for fitting lower fluence transients due to the lack of decay dynamics dependence on the push fluence demonstrated in Figure S3A in the Supporting Information.

Figure 2B demonstrates the effect of such push pulses on the transient photoconductivity of an FA_0.83_Cs_0.17_SnI_3_ thin film for which self-doping had been suppressed by addition of 20% SnF_2_ during the fabrication process.^19^ Here, the sample is photoexcited with a 800 nm wavelength laser pulse (arriving at a nominal delay of −5 ps) which results in generation of free charge carriers and an associated sharp rise in photoconductivity. The transients were measured for different excitation fluences varying between 16 μJ/cm^2^ (red line) and 111 μJ/cm^2^ (blue line), with increased initial free charge-carrier generation leading to an expected increase in photoinduced conductivity. An IR push pulse of 1450 nm wavelength arrives 5 ps later, at nominal time = 0, and causes a distinct feature, a short drop of conductivity, denoted Δ*σ* in the figure. We find that this negative photoconductivity recovers to the original value (before the IR pulse arrival) on subpicosecond time scales. As detailed further below, we suggest that this transient negative conductivity results from THz stimulated emission, generated when free electrons are excited higher into the conduction band by the IR push pulse (or in the case of holes, lower into the valence band), after which they interact with the THz radiation, emitting photons of energy matching those of the THz probe. These “pushed” hot carriers subsequently relax toward the edge of the band, reflected by the time scale of the decay of the transient THz conductivity change. The recovery of the original photoconductivity value also demonstrates that such nonequilibrium charge carriers are retained as free carriers in the conduction band, suggesting an absence of hot-carrier trapping mechanisms that has previously been observed through transient absorption spectroscopy in MAPbBr_3_ and MAPbI_3_ nanocrystals.^39^ We therefore demonstrate that bulk tin iodide perovskite appears to be less susceptible to such detrimental effects, possibly as it offers lower surface-to-volume ratio than nanocrystalline materials. Furthermore, we find that the attribution of the push-induced conductivity drop Δ*σ* to intraband optical excitations of free charge carriers is supported by its magnitude being directly proportional to the density of photoexcited charge-carrier pairs *n*, as shown in Figure 2C. We note that a very small rise of conductivity after the push arrival can be seen even if no pump pulse had arrived prior to the push (black curve in Figure 2B). As detailed in Section V of the Supporting Information, the quadratic dependence of this signal on push fluence suggests an origin in two-photon excitation across the bandgap. This two-photon absorption obscures the dynamics of the photoconductivity transient upon IR pulse arrival. In order to fully account for this effect and to extract the lifetime of the decreased conductivity, a full investigation of the dependence of the two-photon absorption on the push-fluence as well as the initial pump-fluence would need to be performed. Fortunately, as shown in Figure S3 of the Supporting Information and as also visible in Figures 3 and S4, no such two-photon absorption is evident in the highly doped FA_0.83_Cs_0.17_SnI_3_ thin film (no tin fluoride added). This lack of increased conductivity after the IR pulse arrival could be caused by the lower charge-carrier mobility for the highly doped perovskite compared with that of the thin film made with tin-fluoride additive, as well as a blue-shifted absorption spectrum of the doped film resulting from Burstein–Moss effects.^19^ A lower charge-carrier mobility implies a lower conductivity for the same amount of two-photon excitation, and a blue-shifted absorption spectrum may result in reduced IR absorption. We therefore carefully assess the hot-carrier cooling dynamics based on the measurements performed on the highly doped tin-based perovskite (no tin-fluoride additive) as detailed below.

We note that in our pump-push-probe scheme, an ultrafast photoexcitation pulse was used, with photon energy just above the measured bandgap of these materials.^19^ Because of the sufficiently slow charge-carrier recombination dynamics observed in FA_0.83_Cs_0.17_SnI_3_ with 20% SnF_2_ added,^19^ the photoinduced conductivity of the material is almost constant over time scales of the order of a few picoseconds, as evident from Figure 2B. We note that pulsed excitation (“pump”) has benefits in terms of allowing an extraction of charge-carrier mobilities from OPTP; however, an interesting extension of the technique could also be offered by an implementation of alternate excitation, either continuous wave (CW) or pulsed “pump”, with the former resembling solar illumination even more closely.

To examine further such IR-push-induced transitions of free charge carriers within a band, we explore transient conductivity dynamics for a FA_0.83_Cs_0.17_SnI_3_ thin film produced without tin-fluoride addition during deposition. Since this film exhibits a very high conductivity owing to background hole doping (Figure 1), the valence band is already partially filled with holes, obviating the need for initial photoexcitation. We therefore acquired photoconductivity transients with the pump beam blocked, measuring solely the effect of an IR push pulse on the conductivity of the sample (push-probe only). Figure 3 shows that a negative push-induced photoconductivity is observed, whose recovery dynamics are highly similar to those following the push-induced change in pump-induced conductivity of the FA_0.83_Cs_0.17_SnI_3_ film with low doping density discussed above. Through variation of the fluence of the IR push pulse between 13 and 118 μJ/cm^2^, we find the magnitude of the negative photoconductivity to be proportional to the intensity of the IR push pulse, as demonstrated in Section V of the Supporting Information, again indicating a single-photon intraband excitation. Therefore, regardless of the way in which free carriers are generated, whether through photoexcitation or chemical doping, an IR push induces intraband transitions of these charge carriers, leading to a temporal population of nonequilibrium carriers further from the band edge.

We further analyze these transients in order to determine the mechanism of the subsequent relaxation process following the IR-push pulse excitation. We convolute a monoexponential decay function with Gaussian broadening, reflecting the temporal resolution of the system, to fit to the photoconductivity transients (as detailed in Section VI of the Supporting Information). The solid lines in Figure 3 show resulting fits to the data, from which we determine the relaxation time of the pushed holes to be 357 fs ±10 fs for an IR push of the highest fluence (118 μJ/cm^2^) and 1390 nm wavelength (corresponding to 0.89 eV above-gap excess energy). We further find the hot-carrier relaxation dynamics to be push-fluence-independent, as shown in Figure S3A in Section V of the Supporting Information, and fit the lower-fluence data in Figure 3 with our decay model, fixing the relaxation time to that achieved for the highest-fluence transient. The lack of fluence dependence of hot-carrier relaxation dynamics suggests that, unlike in previous studies,^34^ thermalization processes are not dominated by scattering occurring within the freshly generated nonequilibrium hot-carrier distribution. Importantly, such push-fluence independent relaxation times also prove an absence of hot-phonon bottleneck effects, most likely because we employed much lower IR-push fluences here than used in previous investigations of lead-halide perovskites.^37^ We therefore conclude that the energy loss of holes pushed into nonequilibrium is most likely dominated by scattering with the large, thermalized population of holes near the top of the valence band. Any potential contributions from phonon-assisted processes to this thermalization are either negligible or unimpeded by the buildup of a hot-phonon distribution due to relatively low densities of hot carriers, below the hot-phonon bottleneck threshold. Moreover, the dependence of the push-induced signal on the wavelength of the push (discussed further below) suggests that the hot-carrier energy loss predominantly derives from scattering with other charge carriers, rather than phonons, given that the latter would result in a slowing of the cooling dynamics with higher excess energy of the hot carriers, while the opposite is actually observed.^34,35^ We note that the energy loss time we report for this regime is slower than thermalization times for lead halide perovskites under high photoexcitation densities^34^ but orders of magnitude faster than the cooling of a hot charge-carrier gas reported for tin triiodide perovskites following nonresonant excitation.^28−30^ Therefore, a realistic assessment of the usefulness of such materials in hot-carrier extracting solar cells needs to be performed in terms of the time scales required for interaction of charge carriers with an already present background density of cooled charge carriers encountered in a tin-halide perovskite solar cell, owing to background hole doping that arises from tin vacancy formation.

**Figure 4:**
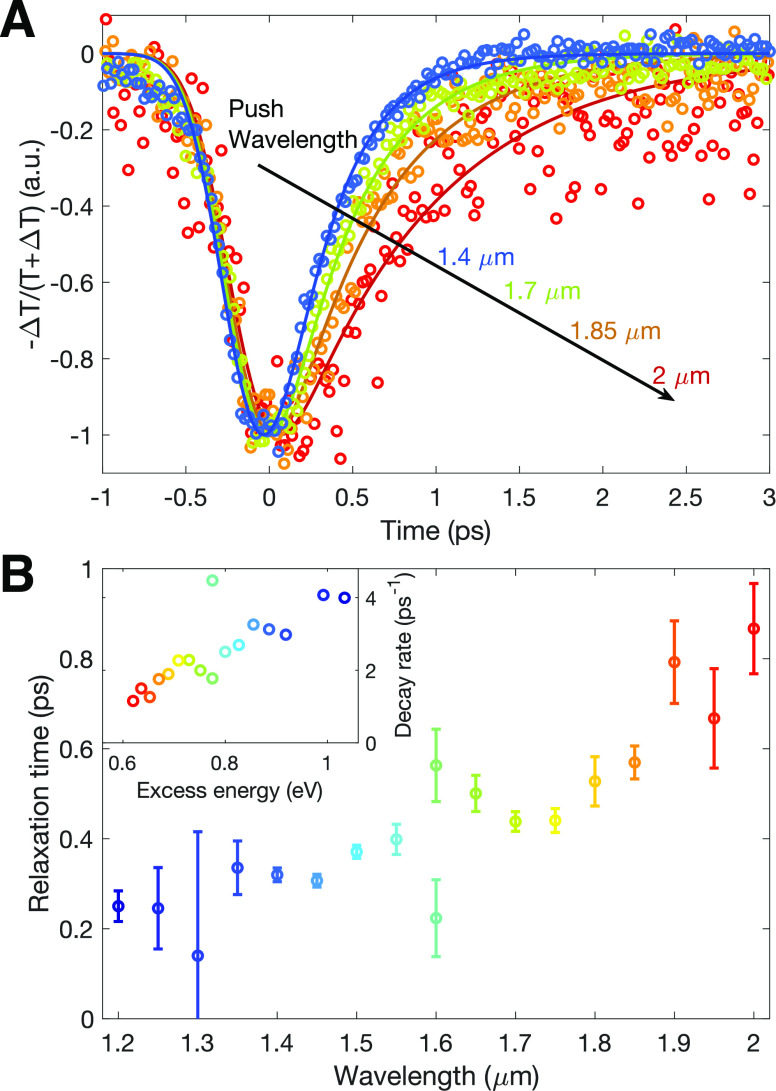
Wavelength dependence of push-induced photoconductivity in a FA_0.83_Cs_0.17_SnI_3_ thin film with high hole doping. (A) Push-induced conductivity transients for a highly doped (no SnF_2_ additive) FA_0.83_Cs_0.17_SnI_3_ thin film, resulting from sub-bandgap IR push pulses of different wavelengths, indicated by the color-coded labels. The data (empty hoops) were scaled to −1 at the peak of the negative response and offset temporarily to time = 0. The darker solid lines represent fits of a monoexponential decay function convoluted with a Gaussian instrument response, used to determine the decay time associated with rethermalization and the strength of the signal. (B) Dependence of charge-carrier relaxation times on push wavelength extracted from fits shown in A and in Figure S4 in the Supporting Information. The error bars represent the 95% confidence intervals of the lifetimes, obtained as the optimal fit parameters. The inset shows the equivalent data, presented instead as charge-carrier scattering rate plotted against photon energy of the push pulse, representing the excess energy imparted on carriers upon the push.

To further elucidate the mechanisms by which nonequilibrium charge carriers relax in the presence of a cooled, already thermalized charge-carrier gas, we investigated how the relaxation dynamics depend on the excess energy provided by the IR push pulse. For this purpose, we monitored the transient push-induced photoconductivity in a highly doped FA_0.83_Cs_0.17_SnI_3_ thin film (no tin fluoride added), while varying the infrared push pulse wavelength using an optical parametric amplifier. Time-dependent photoconductivity transients of these push-probe experiments (analogous to traces shown in Figure 3) are shown in Figure 4A for selected push wavelengths between 1400 and 2000 nm. The full set of decay traces for wavelengths spanning 1200 to 2000 nm is shown in Figure S4 in the Supporting Information. We note that for longer excitation wavelength (lower excess energy), pushed holes relax more slowly toward the top of the valence band compared to the case of excitation with shorter wavelengths (higher excess energy). Figure 4B illustrates the dependence of the relaxation time on the push wavelength, obtained by fitting a convolution of a monoexponential decay with Gaussian broadening to the photoconductivity traces shown here and in Figure S4 in Supporting Information. These fits demonstrate that the lifetime of the hot carriers indeed increases with increased push wavelength (i.e., the relaxation rate increases with increasing excess energy above the band edge, as shown in the inset). This finding is qualitatively consistent with the early thermalization dynamics reported by Richter et al.^34^ for nonequilibrium carriers in lead halide perovskites, who found that higher excess energy increased the average velocity or charge carriers and therefore the carrier–carrier scattering rates. In our case, this higher excess energy imparted upon pushed charge-carriers will therefore lead to faster energy exchange between hot carriers and already thermalized holes at the band edge. The converse effect would be expected to occur if the energy loss of hot carriers were to be dominated by hot-carrier–phonon scattering, which results in slower cooling dynamics being observed for higher excess energies in lead-halide perovskites.^35^ We therefore conclude that for relatively low hot-carrier densities (below the hot-phonon bottleneck threshold), generated in the presence of a large thermalized gas of already cooled charge carriers, a dominant route for dissipation of the excess energy is through scattering of hot charge carriers with the background of cold charge carriers that are already thermalized to the lattice temperature, causing a negligible rise of the carrier gas temperature.

**Figure 5:**
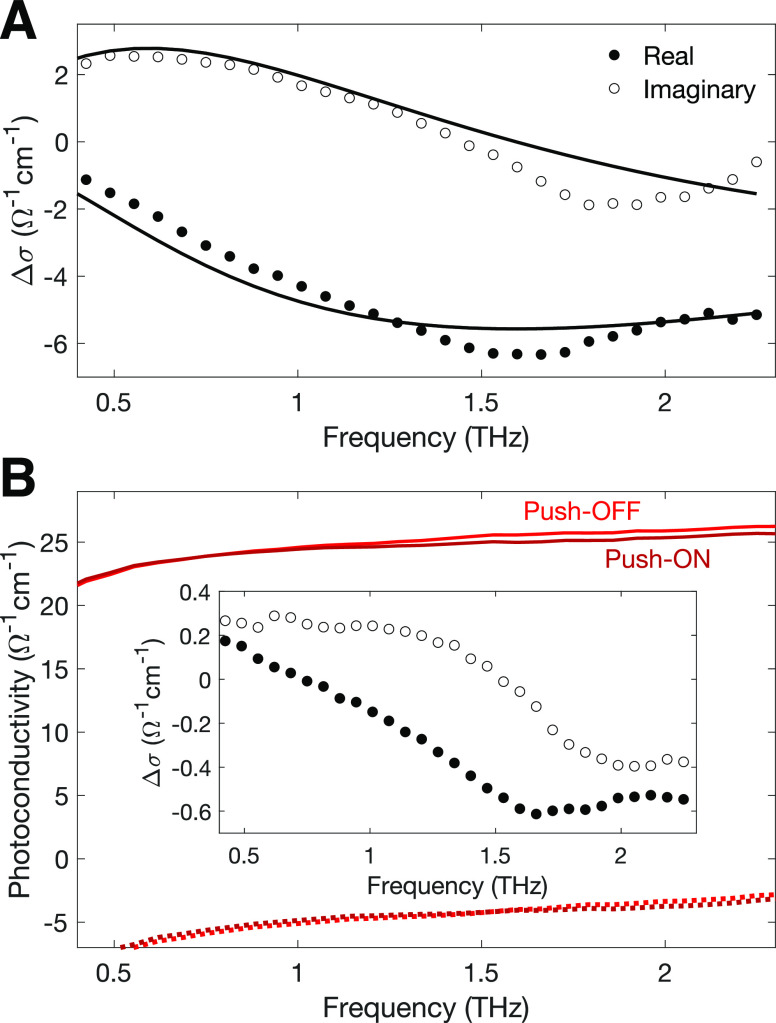
Complex THz photoconductivity spectra for FA_0.83_Cs_0.17_SnI_3_ thin films. (A) Complex push-induced THz conductivity spectrum for highly doped (no SnF_2_ additive) FA_0.83_Cs_0.17_SnI_3_ generated through an IR push pulse only, using a push fluence of 142 μJ/cm^2^. The real part of the push-induced conductivity spectrum is shown by filled circles and the imaginary part by empty hoops. The solid lines represent a negative Lorentzian line shape fit to the measured photoconductivity, which yields a resonant frequency of *f*_0_ = 1.59 THz and spectral broadening of Γ/2π = 3.69 THz, as defined in eq 1. The push-induced conductivity spectrum was measured about 0.1 ps after push pulse arrival. (B) Changes in the pump-induced THz photoconductivity spectra, resulting from the presence of an IR push pulse, for electrically undoped (20% SnF_2_) FA_0.83_Cs_0.17_SnI_3_ photoexcited with a ∼ 40 μJ/cm^2^ pump pulse at 800 nm wavelength. The real part of the photoconductivity is shown by the solid line and the imaginary part by the dotted line. The push-induced difference in the pump- induced photoconductivity, Δ*σ*, is shown in the inset, and calculated from the difference between the Push-ON spectrum (dark red) and Push-OFF spectrum (light red). Filled black circles represent the real part of the push-induced photoconductivity change and empty hoops show its imaginary part. We note that the conductivity transients shown in Figures 2B and 3 represent spectral averages of THz conductivity over the probe’s bandwidth.^51^

Finally, we investigate the origin of the negative THz photoconductivity after sub-bandgap excitation, by examining its frequency dependence across the THz spectral range. A decreased conductivity of a thin film could originate from a decreased density of free charge carriers or a decreased mobility of the existing carriers. Since the THz probe techniques rely on the interaction of the probe photons in the THz frequency range, spectral changes to that interaction (e.g., the emergence of resonant species, or a change of charge-carrier scattering time) also have to be investigated. Finally, as the conductivity is inversely related to the transmission of THz radiation, any mechanisms that enhance the THz radiation (e.g., stimulated THz emission) also have to be considered. To disentangle such effects and determine the mechanism for the observed apparent reduction in THz conductivity upon the arrival of the IR push pulse, we therefore analyze changes in the spectral dependence of the THz photoconductivity (see also further detailed discussion in Section VII of the Supporting Information). Figure 5A shows that the real (solid circles) and imaginary (empty hoops) parts of the THz photoconductivity spectrum of the highly hole-doped FA_0.83_Cs_0.17_SnI_3_ thin film vary with frequency. We find that the spectral shape of the complex push-induced conductivity change Δ*σ* can be described by a negative Lorentz resonance function, given by
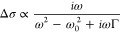
1with the resonant frequency of *f*_0_ = ω_0_/2π = 1.59 THz and broadening parameter Γ/2π = 3.69 THz, as shown in solid black lines in the Figure 5A. We note that a similar negative THz photoconductivity with Lorentzian spectral line shape has been previously associated with stimulated THz emission occurring during photoexcited charge-carrier relaxation in graphene.^44^ Such stimulated THz emission causes an apparent increase of THz transmission which is interpreted as a reduction of conductivity, therefore providing a reasonable explanation for our observations. We therefore conclude that the negative THz conductivity we observe here results from the excitation of free electrons (holes) to energetically higher (lower) states within the conduction (valence) band, following which they interact with the THz probe, releasing stimulated THz emission upon their return to the edge of the band. We note that such stimulated emission processes do not by themselves influence the charge-carrier dynamics provided the probe is sufficiently weak (which we ascertained), similar to the case of the commonly used transient absorption spectroscopy technique based on IR probe pulses.^37^ We further note that although negative THz photoconductivity with similar spectral features can be in principle replicated within the Drude model in association with an increasing effective mass high in a nonharmonic band,^43^ we find this explanation quantitatively incompatible with the very short charge-carrier scattering rates present in perovskite semiconductors, as explained in detail in Section VII of the Supporting Information. Other interpretations of negative photoconductivity present in the literature are specific to low-dimensional materials and rely on e.g. a linear dispersion relation of photoexcited charge carriers in graphene near the Dirac point,^45−47^ which does not apply in our case. We therefore conclude that the change of photoconductivity observed upon push–pulse arrival is neither related to a change in the free-carrier density (previously proposed to result from hot-carrier trapping^39^), nor to changes in the mobility of charge carriers.^43^ These mechanisms would cause a significant change in the DC (0 THz) conductivity that is absent in the data shown in Figure 5. Instead, the interaction of hot carriers with the THz pulse results in stimulated emission of THz photons, increasing the apparent transmission of the probe and leading to a reduction of the apparent THz conductivity, as calculated directly from the transmission intensity of the probe.

In order to verify whether the same mechanism of THz stimulated emission is responsible for the photoconductivity reduction observed in the PPP transients shown in Figure 2B, we measured the photoconductivity spectrum of FA_0.83_Cs_0.17_SnI_3_ with a low level of hole doping (20% SnF_2_ added during film fabrication). To allow determination of just the push-induced spectral component, we measured the complex photoconductivity spectrum shortly after the push arrival with the IR push beam either incident on the sample (“Push-ON” shown in dark red in Figure 5B) or blocked (“Push-OFF” shown in light red). We note that the resulting THz spectra with and without the push present both have features expected from a Drude conductivity model with charge-carrier scattering rate much faster than the frequency of the probing radiation, that is, they exhibit a spectrally flat and positive real part (solid lines) and comparably small imaginary contribution (dotted lines).^48^ This behavior has previously been observed for tin triiodide perovskite thin films for which a charge-carrier scattering time can be estimated to be approximately 1.7 fs.^18^ To determine the conductivity change induced solely by the arrival of the push, we then subtracted the “Push-OFF” photoconductivity spectrum from the “Push-ON” spectrum. This difference, denoted by Δ*σ*, is shown in the inset of Figure 5B and demonstrates the same spectral shape as the push-induced photoconductivity of highly doped (no SnF_2_) FA_0.83_Cs_0.17_SnI_3_, shown in Figure 5A. The similarity of the push-induced spectral changes in THz conductivity between these two experiments (i.e., push-probe for the highly doped film and pump-push-probe for the less doped semiconductor) strongly indicates that a common mechanism is responsible for the decreased conductivity. Therefore, regardless of whether free charge carriers are present through pulsed photoexcitation or whether they are introduced permanently by hole-doping through tin vacancy formation, a push-pulse of sub-bandgap photon energy creates a population of hot charge carriers in thermal nonequilibrium that returns to the band edge accompanied by stimulated THz emission.

In conclusion, we have observed an ultrafast transient reduction in THz conductivity in photoexcited tin-iodide perovskites upon illumination with an infrared push pulse, exploiting a novel Optical-Pump–Infrared-Push–THz-Probe spectroscopic technique. We verify that the same negative photoconductivity dynamics occur in heavily hole-doped tin-iodide perovskite, even in the absence of initial photoexcitation across the band gap, which indicates that they are caused by intraband optical transitions of charge carriers. We further confirm the presence of such intraband, single-photon transitions by investigating the dependence of the conductivity reduction amplitude on the initial free charge-carrier density and push-pulse fluence. We also demonstrate that the spectral shape of the push-induced THz conductivity change is independent of whether the initial free carrier population derives from photoexcitation or chemical doping, confirming a common origin of the transient in intraband transitions of free carriers upon a push. Moreover, the observed Lorentzian line shape and negative value of the push-induced conductivity change suggest the origin of the signal in stimulated THz emission, which enhances the transmitted THz probe as it interacts with pushed charge carriers. We note that previous discussions of negative photoconductivity have focused exclusively on low-dimensional materials such as graphene^44−47^ and doped nanowires.^43^ Therefore, we hope that our discovery of negative photoconductivity in a 3D bulk semiconductor such as tin-iodide perovskite will trigger further discussion about origins of photoinduced conductivity reductions in semiconductors.

Overall, we have demonstrated that the Optical-Pump–IR-Push–THz-Probe technique is a facile and powerful approach toward investigating the relaxation of nonequilibrium charge carriers in perovskite semiconductors in the presence of a cold background charge-carrier density, approximating scenarios found under continuous illumination of sunlight. We find that, in this regime, nonequilibrium charge carriers in tin-iodide perovskites relax to the band edges on subpicosecond time scales. We reveal that such shorter relaxation dynamics are caused by effective scattering of newly generated nonequilibrium charge carriers with an already present density of cooled, thermalized background charge-carriers, as well as lack of a hot phonon bottleneck effect at the low nonequilibrium carrier densities used in our study. While such thermalized, cooled charge-carriers have rarely been present in studies investigating charge-carrier cooling dynamics after nonresonant photoexcitation, they will dominate under continuous illumination conditions encountered in solar irradiation scenarios, especially in highly doped semiconductors. The propensity of tin iodide perovskites toward self-doping arising from facile tin vacancy formation therefore lowers their prospects as candidate materials for hot-carrier solar cells. Overall, we propose that our THz pump–push–probe technique may form an important complementary approach to other pump–push–probe and pump–probe schemes (such as infrared probe and transient absorption measurements)^37,39,40^ used to investigate the relaxation dynamics of nonequilibrium charge carriers. Such approaches will facilitate temporal mapping of the charge-carrier energy exchange and loss dynamics for various background density and excess energy scenarios, which is urgently required for a realistic assessment of the required architecture and the best material contenders for hot-carrier photovoltaic devices.

## Supporting Information Available

Experimental details and sample fabrication method, details of absorption spectrum measurements, photoconductivity spectra analysis, fluence and push-wavelength dependence of transient conductivity (PDF)
