# Supplementary material for: Revealing Ultrafast Charge-Carrier Thermalization in Tin-Iodide Perovskites through Novel Pump–Push–Probe Terahertz Spectroscopy
Source: ACS Photonics. 2021 Aug 9;8(8):2509–18. doi: 10.1021/acsphotonics.1c00763 (PMC8397435; doi:10.1021/acsphotonics.1c00763)
Supplement: Supplementary file 1 — ph1c00763_si_001.pdf [file ph1c00763_si_001.pdf]

Supporting Information: Revealing ultrafast charge-carrier thermalization in tin-iodide perovskites through novel pump-push-probe terahertz spectroscopy

Aleksander M Ulatowski, Michael D Farrar, Henry J Snaith, Michael B Johnston, and Laura M Herz*

Department of Physics, University of Oxford, Clarendon Laboratory, Parks Road, Oxford, OX1 3PU UK

CONTENTS

I. Absorption measurements 2

II. Sample fabrication 3

III. THz Spectroscopy 3

A. Terahertz conductivity measurements 3

B. Pump-Push-Probe spectroscopy 4

IV. Conductivity calculations 5

A. Dark conductivity 5

B. Photoconductivity 5

V. Push-fluence dependence of transients 7

VI. Push-wavelength dependence and decay fitting 9

VII. Investigation of the origin of the negative photoconductivity 11

References 15

* laura.herz@physics.ox.ac.uk

I. ABSORPTION MEASUREMENTS

The measurements of the absorbance of the thin film samples used for estimates of photoexcited charge-carrier density were performed with a Bruker Vertex 80v FTIR spectrometer using a silicon detector and a CaF_2 beam splitter. The transmission and reflection spectra of the thin films are shown in Figure S1. By neglecting scattered light, not accounted for in the reflection/transmission measurement, the fraction of absorbed photons ψ at 800 nm wavelength (wavelength of the pump in photoconductivity experiments) was estimated as $\psi = 1 - R - T$ for both films, obtaining ∼81% photon absorption in $FA_{0.83}Cs_{0.17}SnI_3$ with 20% SnF_2 and ∼58% in the $FA_{0.83}Cs_{0.17}SnI_3$ thin film with no tin-fluoride added.

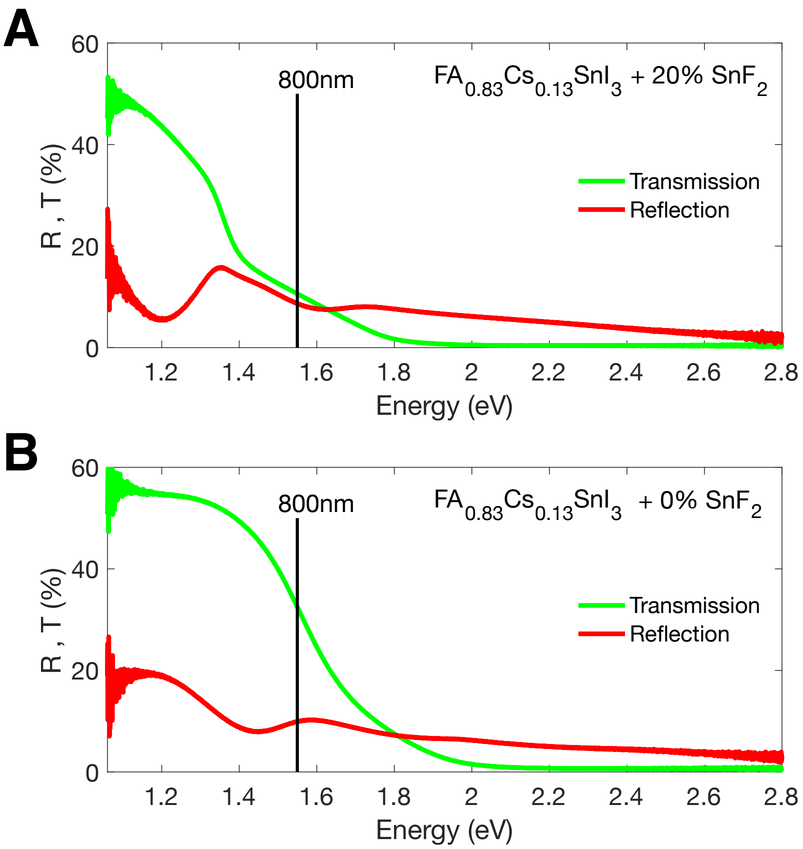

FIG. S1. The fraction of the transmitted (green) and reflected (red) light through thin films of $FA_{0.83}Cs_{0.17}SnI_3$ perovskite with either **A** 20% or **B** 0% SnF_2 added during the fabrication process. The photon energy of the 800 nm wavelength pump pulses used for photoexcitation in the PPP experiments is indicated by a solid black line.

II. SAMPLE FABRICATION

A 1.2 M parent solution of $FA_{0.83}Cs_{0.17}SnI_3$ was prepared 12 hours before sample fabrication, in the mixed solvent of dimethylformamide (DMF) and dimethyl sulfoxide (DMSO) in volumetric ratio of 4:1. 3 mL solution of $FA_{0.83}Cs_{0.17}SnI_3$ was prepared using 513.85 mg of FAI, 159.00 mg of CsI, 1341.07 mg of tin (II) iodide (SnI_2, Alfa Aesar, 99.999% mesh beads), and tin (II) fluoride (SnF_2, Sigma Aldrich) corresponding to 0 or 20 mol% as indicated in the discussion in the main text. Previous studies have demonstrated that the tin fraction in mixed tin-lead perovskites is well controlled by the stoichiometry of the precursor solution, independent of whether SnF_2 additive is used.[1–6]

Z-cut quartz substrates (2 mm thick, 13 mm diameter discs) were prepared by sonicating sequentially in Decon 90 detergent (1% vol. in deionized (DI) water), DI water, acetone and ethanol for 10 min followed by O_2 plasma treatment for 15 min. Films were deposited onto quartz using a single-step antisolvent quenching method in a nitrogen-filled glovebox ($H_2O \leq 0.1$ ppm, $O_2 \leq 1.0$ ppm). 20 μL of the required solution was dispensed onto a static substrate and spun at 4000 rpm with an acceleration of 500 rpm for a total of 60 s. At 35 s, 100μL of anisole was dispensed into the center of the substrate at a height of roughly 5 mm from the surface in a steady stream taking around 1 s to fully dispense. The films turned very dark brown to black instantly. Substrates were then transferred straight onto a hotplate and annealed for 10 min at 100 °C resulting in specular black films. All film preparation parameters were kept constant for each sample. After annealing, all samples were stored in a nitrogen-filled glovebox.

III. THZ SPECTROSCOPY

A. Terahertz conductivity measurements

Terahertz time-domain spectroscopy (THz-TDS) and its extension, Optical-Pump–THz-Probe (OPTP) spectroscopy, have been described previously in multiple publications [7–10]. In an OPTP experiment, the thin-film sample is photoexcited with 800 nm ultrafast ($\sim$35 fs) laser pulse originating from Ti:Sapphire regenerative amplifier system (Spectra Physics: MaiTai-Ascend-Spitfire). The same laser system is used to generate the THz conductivity probe using a multilayer spintronic emitter composed of 2 nm tungsten, 1.8 nm $Co_{40}Fe_{40}B_{20}$,

2 nm platinum (described elsewhere [11]) on a z-cut quartz substrate. The THz probe is focused onto the thin-film sample and subsequently onto the detection crystal with off-axis parabolic gold-coated mirrors. The detection of the THz probe is realised through the electro-optic effect in a 1 mm-thick ZnTe (110) crystal with a 800 nm-wavelength gate originating from the laser setup described above. The change of polarisation of the gate pulse is then measured using a quarter-wave plate, polarising beam cube and balanced photodiode detector. The fluence of the pump beam is controlled with a variable ND filter wheel and the time delay between optical pulses is adjusted with optical delay stages. The differential signal of THz transmission is detected by using optical choppers and lock-in amplifiers as well as a data acquisition board made in-house, and the conductivity of the thin film is calculated using the Tinkham formula [12–16], as detailed in Section IV A.

B. Pump-Push-Probe spectroscopy

An additional infrared "push" pulse of adjustable wavelength was added to the OPTP setup described above in order to perform Optical-Pump–IR-Push–THz-Probe spectroscopy. The IR pulse originates from an optical parametric amplifier (Light Conversion: TOPAS-C), pumped with 800 nm wavelength beam from the Ti:Sapphire laser system described above. The delay of the pulse is controlled with an optical delay stage and its fluence with a variable ND filter wheel. The IR push beam is recombined with the main 800 nm pump using a longpass dichroic beamsplitter with 950 nm-wavelength onset. The wavelength of the push pulse was set to 1450 nm in the pump-push-probe measurement presented in Figure 2B in the main text, and to 1430 nm during the spectral measurement presented in Figure 5B in the main text. The push-probe experiments involving the hole-doped, $FA_{0.83}Cs_{0.17}SnI_3$ thin film fabricated without SnF_2 (Figure 3 and Figure 5A in the main text) were performed with an IR push pulse of 1390 nm wavelength. The estimated fluence of the push pulse was 142 μJ/cm^2 in the spectral push-probe experiment shown in Figure 5A, 254 μJ/cm^2 in the pump-push-probe measurement shown in Figure 2B and 46 μJ/cm^2 in the spectral pump-push-probe measurement shown in Figure 5B of the main text.

IV. CONDUCTIVITY CALCULATIONS

In terahertz time-domain spectroscopy (THz-TDS) as well as in its extensions including the OPTP and pump-push-probe experiments, the transmission of THz probing radiation is measured in order to calculate the conductivity of the sample [15–17]. Such THz transmission can be measured as a function of THz-pulse and gate-pulse delay and then Fourier transformed to yield the frequency-dependent transmission function, used for calculations of dark- and photo-conductivity spectra. Alternatively, the THz transmission can be measured at the time-domain peak of the THz pulse as a function of the pump-probe delay to yield a frequency-averaged transient of photo-induced conductivity change [18].

A. Dark conductivity

The conductivity spectrum of a thin-film sample in the dark (in the absence of any photoexcitation), σ_{dark}, can be calculated from the frequency-dependent transmission through the sample T_{sample}, by comparison with the transmission function through a bare substrate $T_{\text{substrate}}$, according to the commonly used Tinkham formula [12–16],

$$\sigma_{\text{dark}} = \frac{T_{\text{substrate}} - T_{\text{sample}}}{T_{\text{sample}}} \frac{\epsilon_0 c(1 + n_{\text{substrate}})}{L} , \tag{S1}$$

where L is the thickness of the thin film, $n_{\text{substrate}}$=2.1 is the refractive index of the z-cut quartz substrate [19], c is the speed of light and ϵ_0 is the permittivity of free space. Equation S1 was used to calculate the dark conductivity spectra presented in Figure 1 in the main text from the time-domain transmission data shown in the inset of that figure, using L =400 nm as the films' thicknesses, as previously determined for these sets of samples [20].

B. Photoconductivity

In a similar manner, the photo-induced change of conductivity (called just "photoconductivity") can be calculated using the previously discussed formula [18],

$$\sigma_{\text{photoconductivity}} = -\frac{\Delta T}{T + \Delta T} \left[\frac{\epsilon_0 c(1 + n_{\text{substrate}})}{L} \right] \frac{T_{\text{substrate}}}{T} , \tag{S2}$$

where T represents the strength of the transmitted THz field through the sample in the dark and ΔT is the photo-induced change of transmission upon photoexcitation ($\Delta T =$

$T_{\text{photoexcited}} - T_{\text{dark}}$). Equation S2 was used to calculate the spectral conductivities shown in Figure 5 A and B of the main text, with the term $\frac{T_{\text{substrate}}}{T}$ set to 1 when calculating the photoconductivity of $FA_{0.83}Cs_{0.17}SnI_3$ perovskite with 20% SnF_2 included in the fabrication, because of its low dark conductivity and in order to avoid the additional introduction of experimental uncertainties caused by phase errors in the THz-TDS measurements [21].

Because of the proportionality between the photoconductivity and the experimentally determined quantity $-\frac{\Delta T}{T+\Delta T}$, we used this relative change of frequency-averaged THz transmission (measured at the time-domain peak of the THz probe) to illustrate the photoconductivity transients in Figures 2B, 3 and 4A in the main text as well as in Figures S2A and S3A in the SI.

V. PUSH-FLUENCE DEPENDENCE OF TRANSIENTS

The fluence-dependence of the push-induced conductivity change was measured using a variable ND filter wheel in push-probe (no pump) experiments.

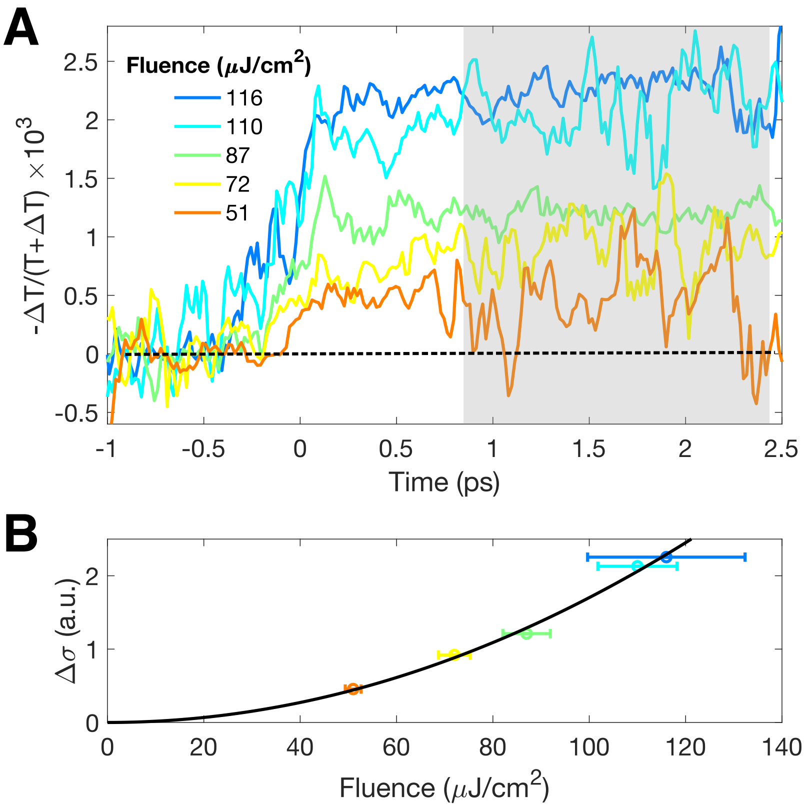

FIG. S2. **A** Positive photoconductivity onset at the push arrival (time=0) measured for a $FA_{0.83}Cs_{0.17}SnI_3$ thin film produced with 20% SnF_2 added during fabrication (low doping level). The fluence of the IR push pulses is indicated in the legend and the greyed-out region was used to obtain the average photoconductivity value $\Delta\sigma$ after the signal onset. **B** The magnitude $\Delta\sigma$ of the photoconductivity after the arrival of the push pulse is plotted against the excitation fluence. The error bars represent the uncertainty of the laser power measurement and the solid black line shows a quadratic fit passing through the origin, i.e. $\Delta\sigma = a \times \text{Fluence}^2$.

Figure S2A shows the photoconductivity onset for a $FA_{0.83}Cs_{0.17}SnI_3$ thin film for which 20% SnF_2 had been added during fabrication (low doping level). The sample was illuminated at time=0 with 1390 nm wavelength IR push pulses. The positive photoconductivity indicates that excitation across the bandgap had occurred, which may be caused by two-photon

excitation. Figure S2B shows the integrated photoconductivity from the greyed-out region of figure S2A as a function of push pulse fluence. The quadratic dependence of the positive photoconductivity on the excitation fluence indeed indicates that a two-photon absorption process is responsible for the across-bandgap charge-carrier excitation [22, 23].

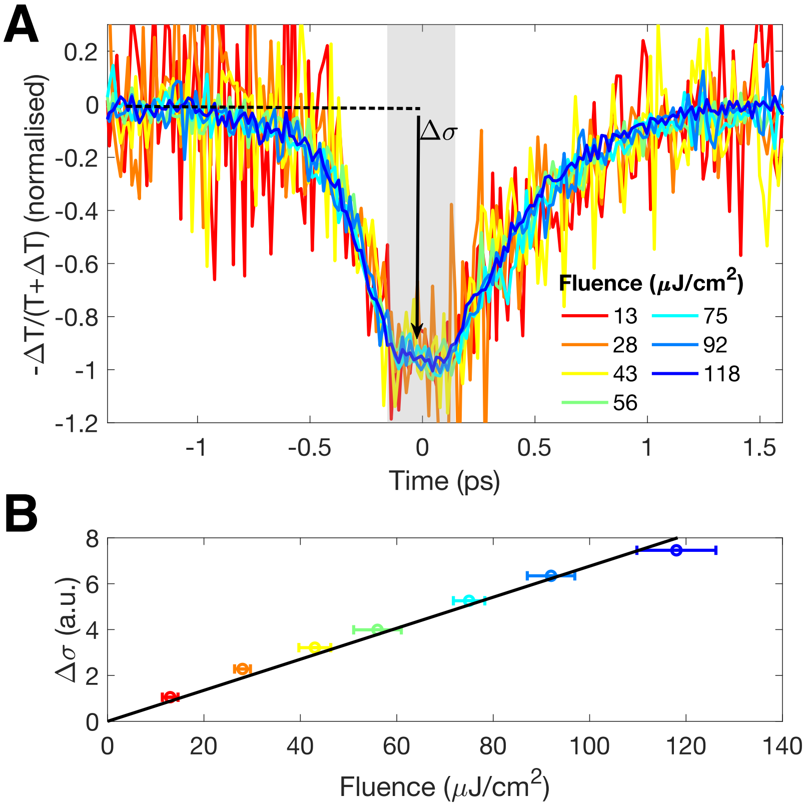

FIG. S3. **A** Negative photoconductivity transients near time=0 (corresponding to the arrival time of the IR push) measured for a $FA_{0.83}Cs_{0.17}SnI_3$ thin film for which no SnF_2 had been added during fabrication (high doping level). Traces were normalised for all fluences to a value of -1 at the peak. The fluence of the IR push beam is indicated by the legend and the magnitude of the push-induced change of conductivity $\Delta\sigma$ was evaluated by averaging the signal strength over the greyed-out region of the unnormalized traces. **B** The magnitude of the photoconductivity $\Delta\sigma$ after push arrival is plotted against the excitation fluence. The error bars represent the uncertainty of the laser power measurement and the solid black line shows a linear fit passing through the origin ($\Delta\sigma = a \times \text{Fluence}$).

In contrast, Figure S3A shows negative photoconductivity transients upon push pulse

arrival for the $FA_{0.83}Cs_{0.17}SnI_3$ thin film for which no SnF_2 had been added during fabrication (high doping level). The transients are normalised to a value of -1 at the peak of the negative photoconductivity (the unnormalized traces are shown in Figure 3 in the main text). The normalization of the fluence-dependent photoconductivity transients reveals that relaxation dynamics are independent of the density of non-equilibrium charge-carriers generated with the IR pulse, justifying the use of the monoexponential decay model (described below) that was implemented with results shown in Figure 3 in the main text. The push-induced change of conductivity $\Delta\sigma$ is evaluated from the unnormalized transients as the average magnitude of the signal around time=0 (grey shaded region in Figure S3A) and plotted in Figure S3B against infrared excitation (push) pulse fluence. The linear dependence of the negative photoconductivity on push fluence indicates a single-photon process causing the conductivity change.

VI. PUSH-WAVELENGTH DEPENDENCE AND DECAY FITTING

The wavelength dependence of the push-induced photoconductivity transient was measured in a push-probe (no pump) experiment using the highly hole-doped thin film of $FA_{0.83}Cs_{0.17}SnI_3$ (no SnF_2).

Figure S4 shows the push-induced photoconductivity traces for excitation wavelengths varied between 1200 nm and 2000 nm. All decay traces of the photoinduced conductivity change $\Delta\sigma$ have been fitted with monoexponential decay curves that were convoluted with a Gaussian broadening representing the temporal resolution of the setup:

$$\Delta\sigma = \left[\exp\frac{-(t+t_{\text{offset}})^2}{2s^2}\right] * \left[a\exp\left(\frac{-t}{\tau}\right)\frac{\text{sgn}(t)+1}{2}\right] \tag{S3}$$

where t_{offset} is an arbitrary time offset to the push pulse arrival, s is the standard deviation of the Gaussian function representing the system time resolution, a is the magnitude of the signal strength and τ is the monoexponential lifetime of the decay. The sign function $\text{sgn}(t)$ has a value of -1 for $t<0$ and +1 for $t>0$, such that the last factor represents the step-like onset of a photoconductivity transient for the case of instantaneous photoexcitation. The temporal resolution of the system s had been first determined as 200 fs by fitting all presented traces with Equation S3 and setting an average resolution from values obtained from these fits. For the presented fitting curves drawn in darker solid lines in Figure S4,

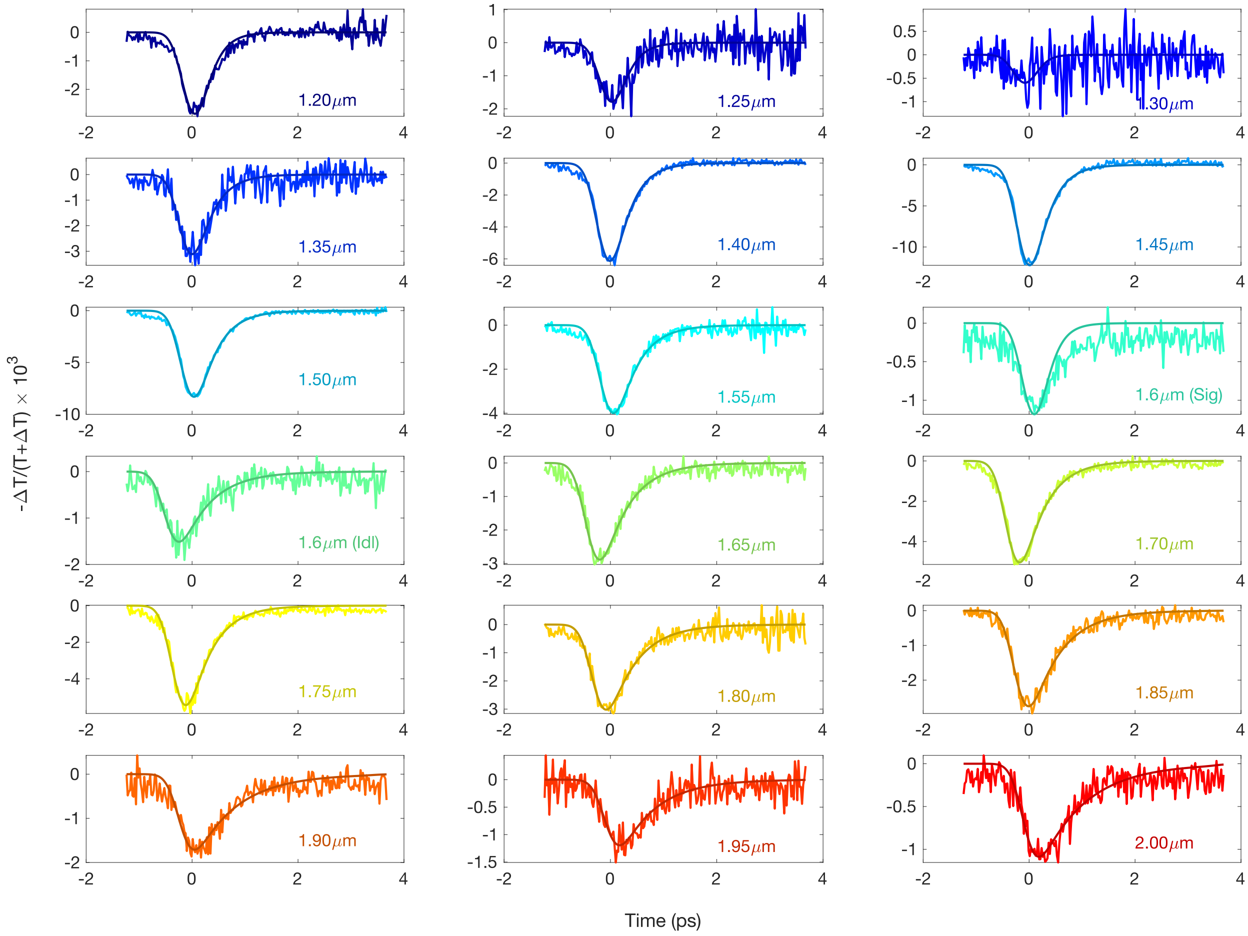

FIG. S4. Negative photoconductivity transients following sub-bandgap IR excitation of a hole-doped thin film of $FA_{0.83}Cs_{0.17}SnI_3$ (no tin fluoride added during fabrication). The wavelength of the IR push pulse was varied between 1200 nm (dark blue) and 2000 nm (red), with the 1600 nm wavelength experiment taken once each for the two possible sources of the pulse (originating either from idler or signal beam of the optical parametric amplifier). The fits according to Equation S3 are shown in darker solid lines on top of the data and the obtained monoexponential decay times of the transients are shown in Figure 4 in the main text.

a fixed value of $s = 200$ fs was therefore used. The same Equation S3 was used for fitting the photoconductivity transients shown in Figure 3 in the main text, with the decay time determined from the fit to the highest fluence data (to minimise the effect of the noise at low fluences) which was then subsequently fixed for all fits to lower-fluence curves.

Figure S5 shows the dependence of the push-induced conductivity decrease (evaluated in terms of the signal strength parameter a, as defined in Equation S3) on the wavelength of

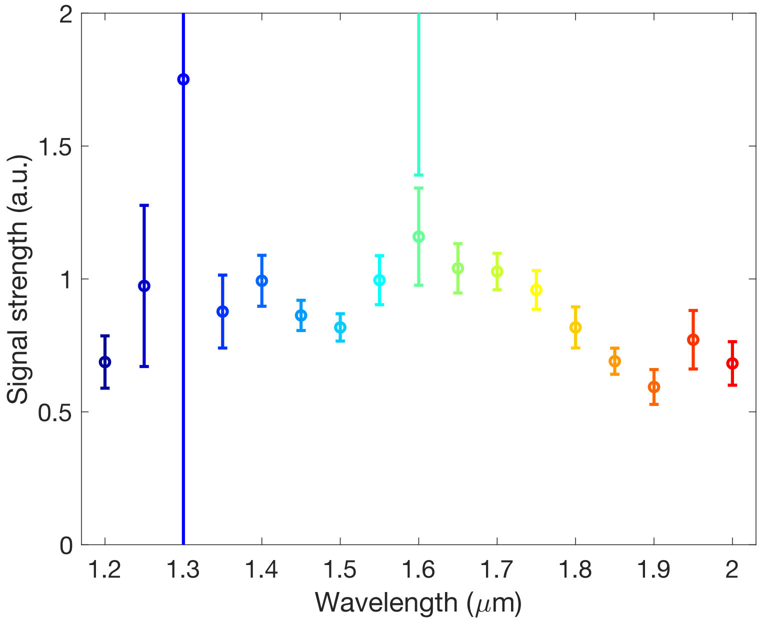

FIG. S5. Magnitude of the negative photoconductivity strength a, defined in Equation S3, normalized by fluence of the push pulse. The error bars represent the uncertainty of the laser power measurement combined with 95% confidence intervals of the fitting parameter obtained from fitting Equation S3 to data shown in Figure S4 .

the push pulse used. The signal strength was normalized by the fluence of the push pulse and shows no strong wavelength dependence compared to the measurement uncertainty.

VII. INVESTIGATION OF THE ORIGIN OF THE NEGATIVE PHOTOCONDUCTIVITY

A light-induced decrease in THz conductivity has previously been observed in low-dimensional doped semiconductors [24–28]. However, existing reports of this negative photoconductivity attributed the phenomenon to a range of different mechanisms, even when the same material was considered [25–28], exposing the lack of agreement in the field about the origin of this effect. In our present study, we investigate thin films of three-dimensional bulk perovskite semiconductors. We therefore consider the proposed mechanisms in the context of the presented data, excluding previously proposed negative photoconductivity origins that are specific to low-dimensional systems [28].

One of the proposed mechanisms resulting in negative photoconductivity is a decrease of charge-carrier scattering rate upon the incidence of light, with simultaneous increase of the charge-carrier effective mass [24]. This effect would result in Drude conductivity that decreases faster with increasing probe frequency as a result of the lowered charge-carrier scattering rate, but remains unchanged for low probe frequencies because the increase of charge-carrier effective mass compensates the increased mobility. The frequency dependence of Drude-like conductivity is described by

$$\sigma_{\mathrm{Drude}}(\omega) = \frac{\sigma_{\mathrm{DC}}}{1 - i\omega\tau}\,, \tag{S4}$$

where τ is the charge-carrier scattering time (or the inverse of the momentum relaxation rate) and σ_{DC} is the low-frequency value of the conductivity. The DC conductivity itself depends on the density of charge carriers n, their effective mass m^* and scattering time: $\sigma_{\mathrm{DC}} = ne^2\tau/m^*$. This model of negative photoconductivity assumes that although the charge-carrier scattering time increases, the overall DC conductivity remains fortuitously unchanged as a result of an exactly compensating rise of effective mass.

Figure S6 shows the simulated Drude conductivity spectra, before (Push-OFF) and after (Push-ON) IR excitation of the thin film. Figure S6A displays in a light-red (Push-OFF) solid line the real part and in a dotted line the imaginary part of a Drude conductivity spectrum for a hybrid-perovskite-like semiconductor. The charge-carrier scattering rate was set to $\tau_{\mathrm{Push-OFF}}$ =1.7 fs as determined previously for tin-iodide perovskites [29]. The calculated conductivity spectrum after IR excitation (Push-ON) is shown in dark-red lines and it features unchanged DC conductivity and increased charge-carrier scattering time. The inset in this figure shows the push-induced change of the conductivity spectra, $\Delta\sigma = \sigma^{\mathrm{Push-ON}} - \sigma^{\mathrm{Push-OFF}}$, with the empty hoops and filled circles representing the imaginary and real parts of the measured push-induced photoconductivity respectively and solid lines showing fits to the data based on the model described above. The charge-carrier scattering time after IR photoexcitation was determined using a least-squares fitting method as $\tau_{\mathrm{Push-ON}} = 0.29\,\mathrm{ps}$. However, such a change in charge-carrier scattering rate would increase the mobility, and therefore the DC conductivity by a factor of $\tau_{\mathrm{Push-ON}}/\tau_{\mathrm{Push-OFF}} = 170$, requiring a rise of the effective mass by the same amount to explain the observed unchanged value of the conductivity at low probe frequencies ($\Delta\sigma \sim 0$ at $\omega = 0$). Figure S6B shows the same calculation as shown in subfigure A, relaxing the condition of the initial scattering

time being set to $\tau_{\mathrm{Push-OFF}}$ =1.7 fs but limiting the relative change of scattering time to 20%. Although this model shows an improved quality of the fits in comparison to data, replicating important spectral features, such as the zero-crossing of the imaginary part, the obtained charge-carrier scattering times are $\tau_{\mathrm{Push-OFF}}$ =65.7 fs and $\tau_{\mathrm{Push-ON}}$ =78.8 fs, which is incompatible with the measured photoconductivity spectra presented in Figure 5B in the main text, which reveal much shorter charge-carrier scattering times (flatter spectral responses). We therefore exclude this explanation as a possibility for the negative photoconductivity in the case of the tin-iodide perovskite thin films investigated here.

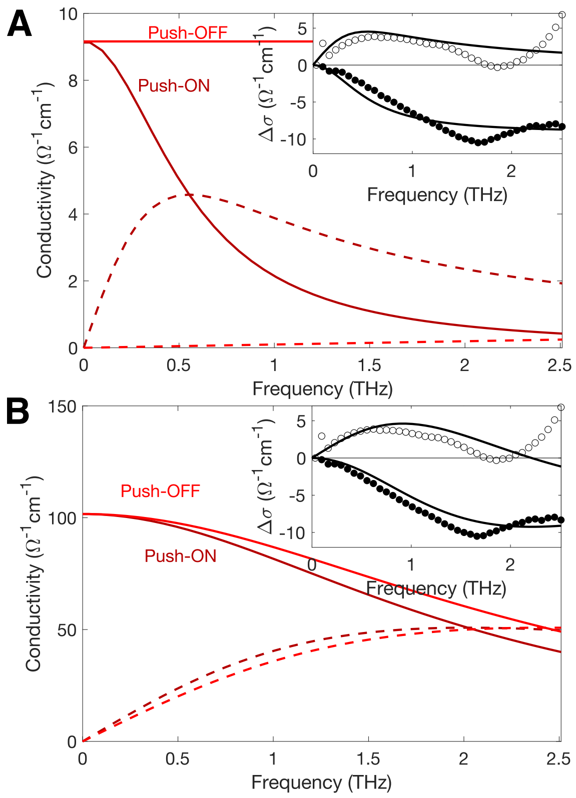

FIG. S6. **A** Conductivity spectrum of a hybrid-perovskite-like semiconductor calculated without the presence of an IR pulse (Push-OFF: light-red lines) and after IR excitation (Push-ON: dark-red lines). The real part of the conductivity is shown in solid and the imaginary part in dotted lines. The spectrum was calculated using Equation S4, setting $\tau = \tau_{\text{Push-OFF}}$ =1.7 fs for the Push-OFF spectrum and leaving σ_{DC} as a free parameter, which was evaluated together with the scattering time of the charge-carriers after IR excitation ($\tau_{\text{Push-ON}}$) by fitting the push-induced change in the conductivity spectra, $\Delta\sigma = \sigma^{\text{Push-ON}} - \sigma^{\text{Push-OFF}}$, to the data shown in the inset. The inset contains the real (filled circles) and imaginary (empty hoops) parts of the photoconductivity spectrum of for $FA_{0.83}Cs_{0.17}SnI_3$ thin film to which no SnF_2 had been added during fabrication (high doping levels), measured in the same way as the spectrum shown in Figure 5A in the main text. The solid black lines show the fit to data, resulting in $\tau_{\text{Push-ON}} = 0.29$ ps and σ_{DC}=9.16 Ω^{-1}cm^{-1}. **B** Calculated conductivity spectra equivalent to those displayed in (A) but with the relative change of charge-carrier scattering time limited to 20%, resulting in an optimal fit for σ_{DC}=101.7 Ω^{-1}cm^{-1}, $\tau_{\text{Push-OFF}}$ =65.7 fs and $\tau_{\text{Push-ON}}$ =78.8 fs.

REFERENCES

---

[1] W. Liao, D. Zhao, Y. Yu, N. Shrestha, K. Ghimire, C. R. Grice, C. Wang, Y. Xiao, A. J. Cimaroli, R. J. Ellingson, *et al.*, Fabrication of efficient low-bandgap perovskite solar cells by combining formamidinium tin iodide with methylammonium lead iodide, J. Am. Chem. Soc. **138**, 12360 (2016).

[2] N. K. Noel, S. D. Stranks, A. Abate, C. Wehrenfennig, S. Guarnera, A.-A. Haghighirad, A. Sadhanala, G. E. Eperon, S. K. Pathak, M. B. Johnston, *et al.*, Lead-free organic–inorganic tin halide perovskites for photovoltaic applications, Energy Environ. Sci. **7**, 3061 (2014).

[3] M. Wei, K. Xiao, G. Walters, R. Lin, Y. Zhao, M. I. Saidaminov, P. Todorović, A. Johnston, Z. Huang, H. Chen, *et al.*, Combining efficiency and stability in mixed tin–lead perovskite solar cells by capping grains with an ultrathin 2D layer, Adv. Mater. **32**, 1907058 (2020).

[4] C. C. Stoumpos, C. D. Malliakas, and M. G. Kanatzidis, Semiconducting tin and lead iodide perovskites with organic cations: phase transitions, high mobilities, and near-infrared photoluminescent properties, Inorg. Chem. **52**, 9019 (2013).

[5] C. Liu, J. Fan, H. Li, C. Zhang, and Y. Mai, Highly efficient perovskite solar cells with substantial reduction of lead content, Sci. Rep. **6**, 1 (2016).

[6] F. Hao, C. C. Stoumpos, R. P. Chang, and M. G. Kanatzidis, Anomalous band gap behavior in mixed sn and pb perovskites enables broadening of absorption spectrum in solar cells, J. Am. Chem. Soc. **136**, 8094 (2014).

[7] C. Wehrenfennig, M. Liu, H. J. Snaith, M. B. Johnston, and L. M. Herz, Charge-carrier dynamics in vapour-deposited films of the organolead halide perovskite $CH_3NH_3PbI_{3-x}Cl_x$, Energy Environ. Sci. **7**, 2269 (2014).

[8] P. Tiwana, P. Parkinson, M. B. Johnston, H. J. Snaith, and L. M. Herz, Ultrafast terahertz conductivity dynamics in mesoporous TiO_2: influence of dye sensitization and surface treatment in solid-state dye-sensitized solar cells, J. Phys. Chem. C **114**, 1365 (2010).

[9] C. Wehrenfennig, G. E. Eperon, M. B. Johnston, H. J. Snaith, and L. M. Herz, High charge carrier mobilities and lifetimes in organolead trihalide perovskites, Adv. Mater. **26**, 1584 (2014).

[10] A. M. Ulatowski, A. D. Wright, B. Wenger, L. R. Buizza, S. G. Motti, H. J. Eggimann, K. J. Savill, J. Borchert, H. J. Snaith, M. B. Johnston, *et al.*, Charge-carrier trapping dynamics in bismuth-doped thin films of $MAPbBr_3$ perovskite, J. Phys. Chem. Lett. **11**, 3681 (2020).

[11] T. Seifert, S. Jaiswal, U. Martens, J. Hannegan, L. Braun, P. Maldonado, F. Freimuth, A. Kronenberg, J. Henrizi, I. Radu, *et al.*, Efficient metallic spintronic emitters of ultrabroadband terahertz radiation, Nat. Photonics **10**, 483 (2016).

[12] M. Tinkham, Energy gap interpretation of experiments on infrared transmission through superconducting films, Phys. Rev. **104**, 845 (1956).

[13] R. Glover III and M. Tinkham, Conductivity of superconducting films for photon energies between 0.3 and $40\,\mathrm{kT_c}$, Phys. Rev. **108**, 243 (1957).

[14] H.-K. Nienhuys and V. Sundström, Intrinsic complications in the analysis of optical-pump, terahertz probe experiments, Phys. Rev. B **71**, 235110 (2005).

[15] J. Lloyd-Hughes and T.-I. Jeon, A review of the terahertz conductivity of bulk and nanomaterials, J. Infrared, Millimeter, Terahertz Waves **33**, 871 (2012).

[16] R. Ulbricht, E. Hendry, J. Shan, T. F. Heinz, and M. Bonn, Carrier dynamics in semiconductors studied with time-resolved terahertz spectroscopy, Rev. Mod. Phys. **83**, 543 (2011).

[17] L. Duvillaret, F. Garet, and J.-L. Coutaz, A reliable method for extraction of material parameters in terahertz time-domain spectroscopy, IEEE J. Sel. Top. Quantum Electron. **2**, 739 (1996).

[18] A. M. Ulatowski, L. M. Herz, and M. B. Johnston, Terahertz conductivity analysis for highly doped thin-film semiconductors, J. Infrared, Millimeter, Terahertz Waves **41**, 1431 (2020).

[19] C. L. Davies, J. B. Patel, C. Q. Xia, L. M. Herz, and M. B. Johnston, Temperature-dependent refractive index of quartz at terahertz frequencies, J. Infrared, Millimeter, Terahertz Waves **39**, 1236 (2018).

[20] K. J. Savill, A. M. Ulatowski, M. D. Farrar, M. B. Johnston, H. J. Snaith, and L. M. Herz, Impact of tin fluoride additive on the properties of mixed tin-lead iodide perovskite semiconductors, Adv. Funct. Mater. **30**, 2005594 (2020).

[21] C. La-o Vorakiat, L. Cheng, T. Salim, R. A. Marcus, M.-E. Michel-Beyerle, Y. M. Lam, and E. E. Chia, Phonon features in terahertz photoconductivity spectra due to data analysis artifact: A case study on organometallic halide perovskites, Appl. Phys. Lett. **110**, 123901 (2017).

[22] Y. He, R. Su, Y. Huang, Y. Zhou, Q. Zhao, J. B. Khurgin, Q. Xiong, and X. Xu, High-order shift current induced terahertz emission from inorganic cesium bromine lead perovskite engendered by two-photon absorption, Adv. Funct. Mater. **29**, 1904694 (2019).

[23] G. Walters, B. R. Sutherland, S. Hoogland, D. Shi, R. Comin, D. P. Sellan, O. M. Bakr, and E. H. Sargent, Two-photon absorption in organometallic bromide perovskites, ACS Nano **9**, 9340 (2015).

[24] M. G. Burdanova, A. P. Tsapenko, D. A. Satco, R. Kashtiban, C. D. Mosley, M. Monti, M. Staniforth, J. Sloan, Y. G. Gladush, A. G. Nasibulin, *et al.*, Giant negative terahertz photoconductivity in controllably doped carbon nanotube networks, ACS Photonics **6**, 1058 (2019).

[25] C. J. Docherty, C.-T. Lin, H. J. Joyce, R. J. Nicholas, L. M. Herz, L.-J. Li, and M. B. Johnston, Extreme sensitivity of graphene photoconductivity to environmental gases, Nat. Commun. **3**, 1 (2012).

[26] S. Kar, D. R. Mohapatra, E. Freysz, and A. K. Sood, Tuning photoinduced terahertz conductivity in monolayer graphene: Optical-pump terahertz-probe spectroscopy, Phys. Rev. B **90**, 165420 (2014).

[27] G. Jnawali, Y. Rao, H. Yan, and T. F. Heinz, Observation of a transient decrease in terahertz conductivity of single-layer graphene induced by ultrafast optical excitation, Nano Lett. **13**, 524 (2013).

[28] P. A. George, J. Strait, J. Dawlaty, S. Shivaraman, M. Chandrashekhar, F. Rana, and M. G. Spencer, Ultrafast optical-pump terahertz-probe spectroscopy of the carrier relaxation and recombination dynamics in epitaxial graphene, Nano Lett. **8**, 4248 (2008).

[29] R. L. Milot, M. T. Klug, C. L. Davies, Z. Wang, H. Kraus, H. J. Snaith, M. B. Johnston, and L. M. Herz, The effects of doping density and temperature on the optoelectronic properties of formamidinium tin triiodide thin films, Adv. Mater. **30**, 1804506 (2018).
